# Terpenes from *Zingiber montanum* and Their Screening against Multi-Drug Resistant and Methicillin Resistant *Staphylococcus aureus*

**DOI:** 10.3390/molecules24030385

**Published:** 2019-01-22

**Authors:** Holly Siddique, Barbara Pendry, M. Mukhlesur Rahman

**Affiliations:** School of Health, Sports and Bioscience, University of East London, Stratford Campus, Water Lane, London E15 4LZ, UK; h.siddique@uel.ac.uk (H.S.); b.pendry@uel.ac.uk (B.P.)

**Keywords:** antimicrobial resistance, multi-drug resistant (MDR), methicillin resistant *Staphylococcus aureus* (MRSA), *Zingiber monatnum*, terpenes, (*E*)-8(17),12-labdadiene-15,16-dial, zerumbol

## Abstract

Bioassay directed isolation of secondary metabolites from the rhizomes of *Zingiber montanum* (Fam. Zingiberaceae) led to the isolation of mono-, sesqui-, and di-terpenes. The compounds were characterized as (*E*)-8(17),12-labdadiene-15,16-dial (**1**), zerumbol (**2**), zerumbone (**3**), buddledone A (**4**), furanodienone (**5**), germacrone (**6**), borneol (**7**), and camphor (**8**) by analysing one-dimensional (1D) (^1^H and ^13^C) and two-dimensional (2D) (COSY, HSQC, HMBC, and NOESY) NMR data and mass spectra. Among these terpenes, compounds **1** and **2** revealed potential antibacterial activity (minimum inhibitory concentrations (MIC) values 32–128 µg/mL; 0.145–0.291 mM)) against a series of clinical isolates of multi-drug resistant (MDR) and Methicillin resistant *Staphylococcus aureus* (MRSA).

## 1. Introduction

Antimicrobial resistance has increasingly become a major public health issue that currently claims 700,000 lives every year. It is predicted that if no action is taken, there will be approximately 10 million deaths each year globally by 2050, which will be more than the predicted number of deaths by cancer [1] and will cause a cumulative 100 trillion USD of economic output due to the rise of drug-resistant infections [1]. The morbidity due to resistant infection has doubled since 2007, which equals the burden of HIV, influenza, and tuberculosis [1]. As antibiotic resistance occurs naturally, its mishandling and misuse in humans and animals accelerate the process of development of resistant infection [2]. Infectious diseases, like pneumonia, tuberculosis, gonorrhoea, and salmonellosis are becoming difficult to treat because the antibiotics that are used to treat them are becoming less effective [2]. The gram-positive bacterium *Staphylococcus aureus* relates to an extensive range of infection of skin and soft tissue, pneumonia, endocarditis, sepsis and bacteremia [3] that causes nosocomial infection (resistant to methicillin and vancomycin). Therefore, it is no doubt important to discover new antibiotics to act against multi-drug resistant (MDR) and Methicillin resistant *Staphylococcus aureus* (MRSA).

Since the accidental discovery of penicillin from *Penicillium notatum*, a huge number of antibiotics have been developed from microbes. However, the development of resistance of existing antibiotics to pathogenic microorganisms necessitates the development of new antibiotics from natural sources, including plants, microbes, and marine resources. Medicinal plants have been under-exploited for antimicrobial drug discovery, although plants are considered as leads for the development of several medicines. Some key examples of plant derived medicines include cardioactive digoxin from *Digitalis lanata* [4], anticancer vincristine and vinblastine from *Catharanthus roseus* [5], analgesic morphine from *Papaver somniferum* [6], antimalarial artemisinin from *Artemisia annua* [7], and antiinflamatory salicin from the bark of the willow tree *Salix alba* L. [8]. There are also significant reports of medicinal plants being used as systemic and topical antimicrobial agents in Ayurvedic [9] and Traditional Chinese Medicine [10], as well as in western herbal medicine [11] due to their self-protection strategy to counter bacteria and fungi in their own environment. Hyperforin isolated from the medicinal plant *Hypericum perforatum* exhibited antimicrobial activity with minimum inhibitory concentrations (MIC) value of 0.1 mg/L against methicillin-resistant *Staphylococcus aureus* (MRSA) and penicillin-resistant variants [12]. A series of new acylphloroglucinol isolated from *Hypericum olympicum* showed highly promising antibacterial activity (MICs 0.50–1 µg/mL) against a series of clinical isolates of MRSA strains [13].

*Zingiber montanum* (Fam. Zingiberaceae), an herbaceous plant that produces a clump of leaves from large rhizomes, is indigenous to Bangladesh, India, Malaysia, Thailand, Indonesia, and Sri Lanka [14]. Traditionally, it has been used for the treatment of asthma, sprains, muscular pain, inflammation, wounds, and as a mosquito repellent, a carminative, and an antidysentery agent [15,16]. *Z. montanum* has been reported to exhibit antioxidant [17], radioprotective [17,18], antiulcer [19], and anti-inflammatory [20] properties. In regard to the phytochemical investigation on *Z. montanum*, a number of monoterpene and sesquiterpene hydrocarbons have recently been reported using gas chromatography-flame ionization detection and gas chromatography-mass spectrometry [21]. The antibacterial, antifungal, allelopathic, and acetylcholinesterase inhibitory activities of these terpenes have also been reported [21]. As part of our research into anti-infective secondary metabolites from Bangladesh medicinal plants, the authors report the bioassay directed isolation and identification of a total of eight terpenes from *Z. montanum* and also their antibacterial activity against a panel of clinical isolates of multi-drug resitant (MDR) and methicillin resistance *Staphycococcus aureus* (MRSA) strains. 

## 2. Results and Discussion

The *n*-hexane, CHCl_3_, and MeOH extracts from the rhizomes of *Z. montanum* were initially screened for antibacterial activity (Table 1) against clinical isolates of MRSA strains. Whilst MeOH extract did not exhibit any activity at a concentration of 512 µg/mL, both n-hexane and CHCl_3_ extracts showed activity against the MRSA strains tested with MICs of 64–256 µg/mL. Vacuum liquid chromatography (VLC) fractionation on active crude extracts, followed by further purification using column chromatography over Sephadex LH20, solid phase extraction (SPE), and/or preparative TLC led to the isolation of seven terpenes (**2**–**8**) from n-hexane extract and two terpenes (**1** and **8**) from the CHCl_3_ extract. Among these compounds, **1** and **2** exhibited promising antibacterial activity against MRSA strains with MICs of 64–128 µg/mL (0.145–0.291 mM).

Compound **1** was isolated as colourless amorphous powder from the CHCl_3_ extract of *Z. montanum*. The molecular formula of **1** was established as C_20_H_30_O_2_ from the [M + H]^+^ at *m*/*z* 303.23122 (calculated for C_20_H_31_O_2_ at 303.23240) in the high resolution of mass spectrometry. The ^1^H-NMR (600 MHz, CDCl_3_, Table 2) spectrum showed the presence of two sets of aldehyde protons (δ_H_ 9.40 and 9.63), one olefinic proton resonating at δ_H_ 6.76 (*J* = 6.6 Hz), exomethylene protons at 4.36 and 4.86, three sets of methyl protons, and a number of peaks for methine and methylene protons. The ^13^C-NMR spectrum showed the presence of a total of 20 carbons including two aldehyde carbons (193.8 and 197.5), an exomethylene (108.1), an olefinic methine (135.0), two aliphatic methines, four quaternary carbons, three methyl carbons, and seven methylene carbons. In HMBC, two sets of methyl protons at δ_H_ 0.82 (δ_C_ 22.1 from HSQC) and δ_H_ 0.89 (δ_C_ 33.7 from HSQC) showed a common ^2^*J* connectivity to a carbon at 33.7 (C-4) and ^3^*J* connection with methylene carbon at δ_C_ 42.0 (C-3; δ_H_ 1.41 and 1.18 from HSQC) and methine carbon at δ_C_ 55.8 (C-5; δ_H_ 1.13 from HSQC). H-5 revealed ^3^*J* HMBC correation to methylene carbons at 42.0 (C-3), 38.1 (C-7; δ_H_ 2.02 and 2.42 from HSQC), methine carbon at 56.6 (C-9; δ_H_ 1.90 from HMQC), and methyl carbon at 14.6 (C-20; δ_H_ 0.72 from HSQC). H-9 exhibited HMBC interactions to C-10 (by ^2^*J*), C-11 (by ^2^*J*), C-12 (160.4 by ^3^*J*; δ_H_ 6.76 from HSQC), C-17 (108.1 by ^3^*J*; δ_H_ 4.36 and 4.86 from HSQC), and C-20 (by ^3^*J*). H-12 showed ^3^*J* HMBC connectivity to C-9, methylene carbon at 39.6 (C-14; δ_H_ 3.41 and 3.46 from HSQC), and aldehydic carbon at 193.8 (C-16; δ_H_ 9.40 from HSQC). The other aldehydic proton at H 9.63 (H-15; C 197.5 from HSQC) showed ^3^*J* HMBC correation to a quaternary carbon at 135.0 (C-13). Accordingly, structure of 1 was confirmed as (*E*)-8(17),12-labdadiene-15,16-dial [22]. The NMR spectra of compound **1** are available in Supplementary Material. This compound has previously reported from *Alpinia chinensis* [22] and *Curcuma heyneana* [23]. This is the first report of its isolation from the genus *Zingiber*.

**Figure 1:**
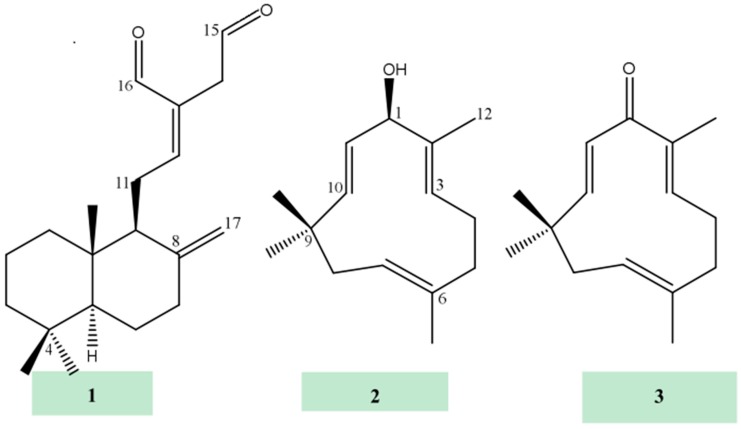

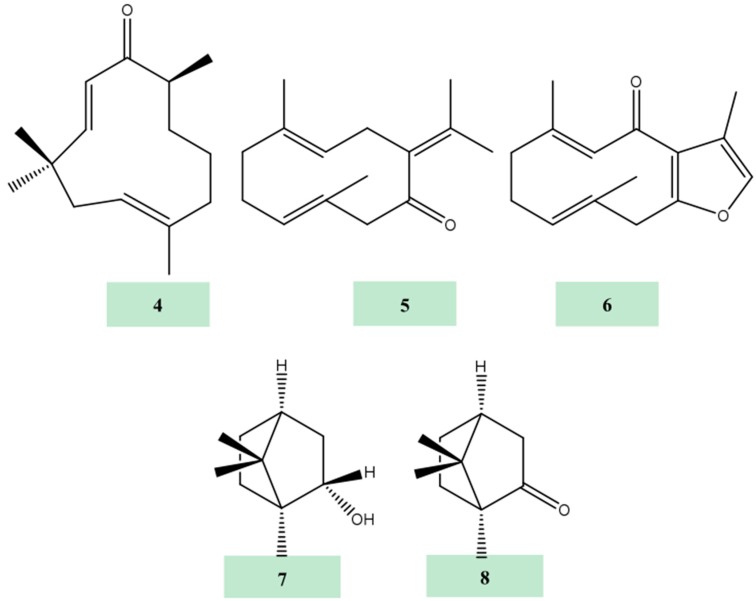
Chemical structures of terpenes isolated from *Z. montanum*.

Compound **2** was isolated colourless oil from the *n*-hexane extract of *Z. montanum*. The IR spectrum revealed the presence of a hydroxyl group (3300 cm^−1^) and double bond (1610 cm^−1^). The high resolution of mass spectroscopy showed the [M + H]^+^ at *m*/*z* 221.18956 (calculated for C_15_H_25_O, at 221.19054), which confirmed the molecular formula of **2** as C_15_H_24_O. The ^1^H-NMR spectrum (CDCl_3_, 600 MHz, Table 3) of **2** showed the presence of four methyl singlets resonating at δ_H_ 1.04, 1.06, 1.43, 1.65, four sets of olefinic protons at 4.82 (dd, *J* = 10.2, 4.4 Hz), 5.20 (d, *J* = 7.5 Hz), 5.23 (d, *J* = 16.2 Hz), and 5.56 (dd, *J* = 16.2, 7.5 Hz), an oxymethine proton at 4.63 as doublet (*J* = 7.5 Hz), and also couple of methylene protons peaks between 1.87–2.35 Hz. The ^13^C-NMR spectrum (150 MHz, CDCl_3_) revealed the presence of a total of 15 carbons, including an oxymethine carbon at 78.8. The DEPT135 identified four methyl, three methylene, one oxymethine, four olefinic methine, and the remaining three as quaternary carbons. Among the later three quaternary carbons, one at 37.3 is aliphatic and remaining two are connected double bonds. The complete structure of this compound was established by two-dimensional (2D) NMR spectra, predominantly by HSQC and HMBC. In the ^1^H-^1^H COSY spectrum, the *trans* double bonded protons showed expected interaction between them. In the HMBC experiment, the *trans* double bonded proton at 5.23 (δ_C_ 139.5 from HSQC) and methyl protons at 1.65 (H-12; δ_C_ 12.8 from HSQC) revealed a common ^3^*J* interaction to an oxymethine carbon at 78.8 (C-1). Olefinic protons at δ_H_ 4.82 (H-7; δ_C_ 125.0 ppm from HSQC) and 5.20 (H-3; δ_C_ 124.8 ppm from HSQC) and methyl protons at 1.43 (H-13; δ_C_ 15.3 ppm from HSQC) showed ^3^*J* correlation to a methylene carbon at 39.5 (C-5; δ_H_ 2.35 ppm from HSQC). Protons at 2.20 and 2.24 (H-4; δ_C_ 24.4 from HSQC) exhibited ^3^*J* correlations to quaternary carbons at 142.2 (C-2) and 133.2 (C-6). Two sets of methyl protons at 1.04 (H-14; δ_C_ 24.9) and 1.06 (H-15; δ_C_ 29.9) revealed common ^2^*J* correlation to a quaternary carbon at 37.3 (C-9) and ^3^*J* interaction to methine carbon at 139.5 (C-10, δ_H_ 5.23) and methylene at 42.4 (C-8, δ_H_ 1.87, 2.32). H-10 also revealed ^3^*J* interaction to both methyl group carbons at 24.9 (C-14) and 29.9 (C-15). The COSY experiment exhibited usual interaction (H-10 to H-11; H-4 to both H-3 and H-5; H-7 to H-8). Accordingly, compound **2** was identified as (2*Z*,6*Z*,10*E*)-2,6,9,9-tetramethylcycloundeca-2,6,10-trien-1-ol, commonly known as zerumbol (**2**) [24]. The NMR spectra of compound **2** are available in Supplementary Material. Compounds **3**–**8** were identified as zerumbone (**3**) [23,25], buddledone A (**4**) [26], germacrone (**5**) [23,27], furanodienone (**6**) [28], borneol (**7**) [29], and camphor (**8**) [29]. Among these compounds, zerumbol (**2**), buddledone A (**4**), germacrone (**5**), and furanodienone (**6**) have been reported first time from the genus *Zingiber*, while zerumbone (**3**) was reported from *Z. zerumbet* [30]. Chemical structures of compounds **1**–**8** are incorporated in Figure 1.

Compounds **1**–**8** were assessed for their antibacterial activities against multi-drug resistant and methicillin resistant *Staphylococcus aureus*, notably SA1199B, XU212, RN4229, EMRSA15, MRSA27819, and MRSA340702. The minimum inhibitory concentrations (MICs) of these compounds are presented in Table 4. Norfloxacin was used as positive control for the comparison of antibacterial potencial of these compounds. Among these compounds, **1** and **2** displayed the highest activities with MICs in the range of 32–128 µg/mL (0.145–0.291 mM) against the test organisms. Compound **1** is a labdane diterpene with exomethylene at C-8, an olefine at C-12, and two aldehyde groups at C-16 and 17. The presence of these groups and unsaturations could account for significant antibacterial activity against MRSA strains. Although compounds **2** and **3** are structurally very similar, they differ in activity. We suggest that the presence of a hydroxyl group instead of carbonyl group at C-1 might make compound **2** more active than compound **3**. The antibacterial activity of compounds **3**–**8** were above 128 µg/mL (0.557–0.842 mM), the highest concentrations at which the compounds were tested. Monoterpene and sesquiterpenes from *Zingiber* were reported with antibacterial activity against *Staphylococcus aureus* (MTCC 96) and *Staphylococcus epidermidis* (MTCC 435) [21].

## 3. Material and Methods

### 3.1. General

Analytical solvents, such as *n*-hexane, chloroform, ethyl acetate, acetone, and methanol were purchased from Fisher Scientific, Loughborough, UK. Dimethyl sulfoxide, sodium chloride, norfloxacin and 3-[4,5-dimethylthiazol-2-yl]-2,5-iphenyltetrazolium bromide (MTT) used during antibacterial assay were purchased from Sigma Aldrich (Dorset, UK). Silica gel 60H used for vacuum liquid chromatography was purchased from Merck Millipore, UK. Sephadex LH 20 used for gel filtration chromatography was purchased from GE healthcare, Uppasala, Sweden. Prepacked silica column (normal phase) used for solid phase extraction (SPE) was purchased from Phenomenex, Cheshire, UK. Analytical and preparative TLC carried out on 0.2 mm silica gel 60 F_254_ was purchased from Merck, Darmstadt, Germany. Spots on the TLC plates were visualized under short UV (254 nm) and long UV (366 nm), and also by spraying them with 1% vanillin in concentrated H_2_SO_4_ followed by heating at 100 °C for 3–6 min. The NMR spectroscopy was performed with Bruker AMX 600 NMR spectrometer, Coventry, UK (600 MHz for ^1^H, and 150 MHz for ^13^C) in the Department of Chemistry at University College London (London, UK). High Resolution Mass Spectrometry (HRMS) was performed in Liverpool John Moores University (Liverpool, UK). IR spectroscopy was recorded on Agilent FT-IR (Cary 630, Stockport, UK). 

### 3.2. Plant Material

The rhizomes of *Zingiber montanum* were collected from Bangladesh National Botanical Garden, Dhaka, Bangladesh in September 2016. The plant was identified by the Bangladesh National Herbarium, Mirpur, Dhaka, Bangladesh, where a voucher specimen (DACB 43550) of this collection was deposited.

### 3.3. Extraction and Isolation of Compounds

The rhizomes of *Z. montanum* were sun dried for 2–3 days, followed by drying in the oven at a temperature of 30–35 °C for 30 min prior to grinding. Subsequently, the plant materials were ground into fine powders using a grinder. The ground plant material (242 g) was Soxhlet extracted with solvents of increasing polarity: *n*-hexane, chloroform, and methanol (approximately 700 mL, 10–15 cycles each). Each of the extracts was concentrated using rotary evaporator under reduced pressure at a maximum temperature of 40 °C to yield 9.38 g, 10.22 g, and 23.0 g of *n*-hexane, chloroform, and methanol extracts, respectively. The antibacterial screening was performed on these crude extracts against clinical isolates of MRSA strains. Hexane (MICs 128–256 µg/mL) and chloroform (MICs 64–256 µg/mL) extracts appeared to be active and they were further fractionated by vacuum liquid chromatography (VLC). A portion of *n*-hexane (6.5 g) or chloroform (7.6 g) were adsorbed into silica gel (70–230 mesh) and loaded into VLC column, which was uniformly packed with VLC grade silica gel (60H), followed by eluting with stepwise gradient of mobile phase initially with mixture of *n*-hexane and ethyl acetate and then with EtOAc and MeOH mixtures. The eluted fractions (200 mL each) were evaporated using rotary evaporator and analysed by TLC. Based on TLC results, the similar fractions were bulked together for further purifications by solid phase extraction (SPE), column chromatography over Sephadex LH20, and/or preparative TLC. The basic principle of SPE is similar to VLC, but SPE was used in smaller scale fractionation or further purification of compounds from the VLC fractions or pooled fractions from Sephadex LH20 column chromatography. For column chromatography over Sephadex LH20, the glass column was packed with the slurry of Sephadex LH-20, which was soaked in the solvent (50% chloroform in *n*-hexane or 100% chloroform) half an hour prior to the packing of the column. The sample was dissolved in a small amount of appropriate solvent and then applied onto the top of the adsorbent. The column was eluted with 50–75% chloroform in *n*-hexane, followed by 100% chloroform and then CHCl_3_ + MeOH mixtures of increasing polarity. During preparative TLC, the sample was applied uniformly as band in the sample application zone (2 cm above from the bottom edge of TLC plate) on commercially available TLC aluminium plates (pre-coated silica gel 60 PF_254_). The TLC plates were developed with appropriate mobile phase up to the upper edge of plates. In addition, the multiple development technique was also adapted for a better accomplishment of separation of compounds of very similar polarity.

VLC fraction eluted with 5–10% of EtOAc in *n*-hexane of *n*-hexane extract was subjected to column chromatography over Sephadex LH20. PTLC (mobile phase 15% EtOAc in hexane) on Sephadex column eluted with 50% CHCl_3_ in *n*-hexane yielded compounds **2** (3 mg) and **3** (10 mg), whereas compound **4** (9 mg) was obtained from sephadex column eluted with 100% chloroform. SPE on the VLC fraction eluted with 15% of EtOAc in *n*-hexane of *n*-hexane extract provided six sub-fractions. Preparative TLC (mobile phase 4% EtOAc in hexane plus two drops glacial acetic acid) on SPE sub-fraction eluted with 4% EtOAc in hexane yielded compounds **5** (7 mg) and **6** (4 mg), whilst preparative TLC (mobile phase 4% EtOAc in hexane plus two drops glacial acetic acid) on SPE sub-fraction eluted with 4% EtOAc in hexane led to the isolation of compounds **6** (5 mg) and **7** (7 mg). Similarly, VLC fraction of chloroform extract was subjected to SPE and preparative TLC for the purification of compounds. SPE on VLC fraction eluted with 15% EtOAc in *n*-hexane followed PTLC (mobile phase 4% EtOAc in hexane) on SPE sub-fraction eluted with 4% EtOAc in hexane yielded **8** (4 mg), while compound **1** (6.5 mg) was isolated from the VLC fraction eluted with 25% EtOAc in *n*-hexane, followed by PTLC (mobile phase 15% EtOAc in hexane) on SPE sub-fraction eluted with 10% EtOAc in hexane.

### 3.4. Antibacterial Assay against Clinical Isolates of Multi-Drug Resistant and Methicillin Resistant Staphylococcus Aureus

The antibacterial activity of crude extracts and the isolated compounds were tested against clinical isolates of MRSA strains by microtitre assay using 96 well plates to determine the minimum inhibitory concentrations (MICs). Mueller–Hinton broth (MHB) used in this study was purchased from Oxoid, Hamshire, UK and prepared as instructed by the supplier; however, MHB was adjusted to contain 20 mg/L and 10 mg/L of Ca^2+^ and Mg^2+^, respectively. The clinical isolates of *S. aureus* strains used in this study included ATCC25923, SA1199B, RN4220, XU212, EMRSA15, MRSA340702, and MRSA274829. A standard laboratory strain, ATCC25923, was also used in this study, which is sensitive to antibiotics, like tetracycline [31]. SA1199B over-expresses the NorA MDR efflux pump [32], RN4220 possesses the MsrA macrolide efflux protein [33], XU212 is a Kuwaiti hospital isolate that is an MRSA strain possessing the TetK tetracycline efflux pump [31], whilst the EMRSA15 strain [34] is epidemic in the UK. All *S. aureus* strains were subcultured on nutrient agar (Oxoid) and incubated for approximately 24 h at 37 °C prior to MIC determination. All of the bacterial strains were prepared in 9 g/L saline water with an inoculum density of 5 × 10^5^ colony forming unit (cfu/mL) by comparison with the 0.5 MacFarland turbidity standard.

The stock solution of control positive (Norfloxacin) was prepared by dissolving the antibiotic (2.0 mg) in DMSO (244 µL) and diluting 16 fold with MHB to obtain the desired concentration of the antibiotic stock solution (512 µg/mL). Similarly, stock solutions of crude extract (2048 µg/mL) and isolated compounds (256–512 µg/mL) were prepared by dissolving in required amount of DMSO, followed by 16 fold dilution with MHB.

During the experiment, using a multi-channel pipette an aliquot of 100 µL of MHB was dispensed into each well of 96-well plate except those in the last column. Then 100 µL of stock solution of crude extract or isolated compounds and antibiotic was added in duplicate to the wells of the first column of 96-well plate (total content 200 µL), followed by mixing the content thoroughly and transferring 100 µL of this content to the wells of the second column of 96-well plate using a multi-channel pipette. This two-fold serial dilution process was continued to the 10th well, followed by the addition of the final 100 µL solution to the empty wells of 12th column of 96 well-plate. The inoculum (100 µL) of each bacterium at a density of 5 × 10^5^ cfu/mL was added to all wells, except those in the final (12th) column. The contents of the wells in the 11th and 12th columns represented growth control (bacteria, but no antibiotic, extract, or compounds) and sterility control (antibiotic, extract, or compounds but no bacteria), respectively. Every assay was performed in duplicate. The plates were incubated for 18 h at 37 °C. For the measurement of MIC, 20 µL of a 5 mg/mL methanolic solution of 3-[4,5-dimethylthiazol-2-yl]-2,5-iphenyltetrazolium bromide (MTT; Sigma) was added to each of the wells, followed by incubation for 20–30 min at 37 °C. Bacterial growth was indicated by a colour change from yellow (colour of MTT) to dark blue. The MIC was recorded as the lowest concentration at which no growth (yellow color) was observed [13]. If no growth was observed at any of the concentrations tested, the assay was repeated starting from a stock solution of lower concentration. If growth was observed at all of the concentrations tested, the assay was repeated, starting with a stock solution of higher concentration.

## 4. Conclusions

In this study, the crude extracts of the rhizomes of *Z. montanum* and compounds that were isolated from active extracts were assessed against a panel of clinical isolates of multi-drug resistant (MDR) and methicillin resistant *Staphylococcus aureus* (MRSA), including SA1199B, XU212, RM4221, EMRSA15, MRSA27819, and MRSA340702. Bioassay directed isolation using a range of chromatographic techniques, including vacuum liquid chromatography (VLC), solid phase extraction (SPE), column chromatography over Sephadex LH20, and preparative thin layer chromatography (PTLC) led to the identification of two monoterpenes, borneol (**7**) and camphor (**8**), and five sesquiterpnes, zerumbol (**2**), zerumbone (**3**), buddledone A (**4**), furanodienone (**5**), germacrone (**6**), and a diterpene, (*E*)-8(17),12-labdadiene-15,16-dial (**1**). Among these terpenes, compounds **1** and **2** displayed significant activity with MICs of 32–128 µg/mL (0.145–0.291 mM) against the clinical isolates of MRSA strains tested. Such activity encourages the authors to carry out bioassay guided phytochemical investigation on related members of Zingiberacae family for the identification of lead anti-Staphylococcal compounds.

## Figures and Tables

**Table 1:** Antibacterial activity of crude extracts against standard, multi-drug resistant (MDR) and methicillin-resistant strains of *Staphylococcus aureus* in µg/mL.

Extracts/Antibiotic	MICs in µg/mL				
	SA1199B	XU212	EMRSA15	RN4229	ATCC25941
*n*-Hexane	128	128	256	256	128
Chloroform	64	128	128	128	256
Methanol	>512	>512	>512	>512	>512
Norfloxacin	32	64	16	8	16

**Table 2:** ^1^H- (600 MHz), ^13^C- (150 MHz) NMR and HMBC (600 MHz) data of **1** in CDCl_3_.

Position	1H	13C	HMBC	
			^2^ *J*	^3^ *J*
1	1.06, m, 1H; 1.68, m, 1H	39.4	-	C-9
2	1.50, m, 1H; 1.57, m, 1H	19.5	C-3	C-4
3	1.18, m, 1H; 1.41, m, 1H	42	-	C-18, C19
4	-	33.6	-	-
5	1.13, m, 1H	55.8	C6	C3, C7, C9, C19, C20
6	1.34, m, 1H; 1.75, m, 1H	24.4	C5, C7	C10
7	2.02, m, 1H; 2.42, m, 1H	38.1	C8	C5, C17
8	-	148.4	-	-
9	1.90, m, 1H	56.6	C10, C11	C12, C17, C20
10	-	39.8	-	-
11	2.31, m, 1H; 2.49, m, 1H	24.8	C9	C8, C13
12	6.76, t, *J* = 6.6 Hz, 1H	160.4	-	C9, C14, C16
13	-	135	-	-
14	3.41, d, *J* = 16.8 Hz, 1H	39.6	C13	C12, C16
	3.46, d, *J* = 16.7 Hz, 1H			
15	9.63, t, *J* = 14.4 Hz, 1H	197.5	C14	C13
16	9.40, s, 1H	193.8	C13	C12, C14
17	4.36, s, 1H; 4.86, s, 1H	108.1	C8	C7, C9
18	0.88, s, 3H	33.7	-	C3, C5, C19
19	0.82, s, 3H	22.1	-	C3, C5, C18
20	0.72, s, 3H	14.6	C10	C5, C9

**Table 3:** ^1^H- (600 MHz) and ^13^C- (150 MHz) NMR and HMBC (500 MHz) data of **2** in CDCl_3_.

Position	1H	13C	HMBC	
			^2^ *J*	^3^ *J*
1	4.63, d, *J* = 7.5 Hz, 1H	78.8	-	C3, C10, C12
2	-	142.2	-	-
3	5.20, d, *J* = 7.5 Hz, 1H	124.8	-	C1, C12
4	2.20, m, 1H; 2.24, m, 1H	24.4	C3	C6
5	2.35, m, 2H	39.5	-	C3, C13
6	-	133.2	-	-
7	4.82, dd, *J* = 10.2, 4.4 Hz, 1H	125	-	C5, C9
8	1.87, m, 1H; 2.32, m, 1H	42.4	C7	C6, C10
9	-	37.3	-	-
10	5.23, d, *J* = 16.2 Hz, 1H	139.5	-	C1, C14, C15
11	5.56, dd, *J* = 16.2, 7.5 Hz, 1H	131.2	C1	C9, C13
12	1.65, s, 3H	12.8	C2	C1, C3
13	1.43, s, 3H	15.3	C6	C5, C7
14	1.04, s, 3H	24.9	C9	C8, C10, C15
15	1.06, s, 3H	29.9	C9	C8, C10, C14

**Table 4:** Minimum inhibitory concentrations (MICs) (in mM) of compounds (**1**–**8**) against standard, multi-drug resistant (MDR) and methicillin-resistant strains of *Staphylococcus aureus*.

Compound	SA1199B	XU212	ATCC25941	RN4220	EMRSA15	MRSA27819	MRSA340702
**1**	0.212	0.424	0.212	0.212	0.212	0.424	0.424
**2**	0.291	0.582	0.582	0.582	0.145-0.291	0.582	>0.582
**3**	>0.587	>0.587	>0.587	>0.587	>0.587	>0.587	>0.587
**4**	>0.582	>0.582	>0.582	>0.582	>0.582	>0.582	>0.582
**5**	>0.587	>0.587	>0.587	>0.587	>0.587	>0.587	>0.587
**6**	>0.557	>0.557	>0.557	>0.557	>0.557	>0.557	>0.557
**7**	>0.831	>0.831	>0.831	>0.831	>0.831	>0.831	>0.831
**8**	>0.842	>0.842	>0.842	>0.842	>0.842	>0.842	>0.842
Norfloxacin	0.100	0.200	0.050	0.025	0.050	0.100	0.401

## Supplementary Materials

NMR and mass spectra of active compounds together with results of antibacterial activity using 96 well plates are available in supplementary materials.
