# Supplementary material for: Terpenes from Zingiber montanum and Their Screening against Multi-Drug Resistant and Methicillin Resistant Staphylococcus aureus
Source: Molecules. 2019 Jan 22;24(3):385. doi: 10.3390/molecules24030385 (PMC6384810; doi:10.3390/molecules24030385)
Supplement: Supplementary file 1 [file molecules-24-00385-s001.pdf]

Supplementary material

Terpenes from *Zingiber montanum* and Their Screening against Multi-Drug resistant and Methicillin resistant *Staphylococcus aureus*

Holly Siddique, Barbara Pendry, and M. Mukhlesur Rahman*

School of Health, Sports and Bioscience, University of East London, Stratford Campus, Water Lane, London E 15 4LZ, UK

* Correspondence: email: m.rahman@uel.ac.uk; Tel.: +44-2082234299

Contents

1. ^{1}H and ^{13}C NMR assignments of compounds **1** p S3-S3
2. ^{1}H, ^{13}C, COSY, HSQC and HMBC NMR spectra of compound **1** p S4-S9
3. HRMS spectrum of compound **1** p S10-S10
4. ^{1}H and ^{13}C NMR assignments of compounds **2** p S11-S11
5. ^{1}H, ^{13}C, COSY, HSQC and HMBC NMR spectra of compound **2** p S12-17
6. HRMS spectrum of compound **2** p S18-S18
7. Photographic image of antibacterial activity (MICs in mg/L) of compounds 1-3 and norfloxacin p S19-19

^{1}H and ^{13}C NMR spectra of compound 1:

1.06 (m, 1H, H-1a), 1.68 (m, , 1H, H-1b), 1.50 (m, 1H, H-2a), 1.57 (m, 1H, H-2b), 1.18 (m, 1H, H-3a), 1.41 (m, 1H, H-3b), 1.13 (m, 1H, H-5), 1.34 (m, 1H, H-6a), 1.75 (m, 1H, H-6b), 2.02 (m, 1h, H-7a), 2.42 (m, 1H, H-7b), 1.90 (m, H-9), 2.31 (m, 1H, H-11a), 2.49 (m, 1H, H-11b), 6.76 (t, *J*= 6.6 Hz, 1H, H-12), 3.41 (d, *J*=16.8 Hz, 1H, H-14a), 3.46 (d, *J*=16.7 Hz, 1H, H-14b), 9.63 (t, *J*=14.4 Hz, 1H, H-15), 9.40 (s, 1H, H-16), 4.36 (s, 1H, H-17a), 4.86 (s, 1H, H-17b), 0.89 (s, 3H, H-18), 0.82 (s, 3H, H-19) and 0.72 (s, 3H, H-20).

39.4 (C-1), 19.5 (C-2), 42.0 (C-3), 33.6 (C-4), 55.8 (C-5), 24.4 (C-6), 38.1 (C-7), 148.4 (C-8), 56.6 (C-9), 39.8 (C-10), 24.8 (C-11), 160.4 (C-12), 135.0 (C-13), 39.6 (C-14), 197.5 (C-15), 193.8 (C-16), 108.1 (C-17), 33.7 (C-18), 22.1 (C-19) and 14.6 (C-20).

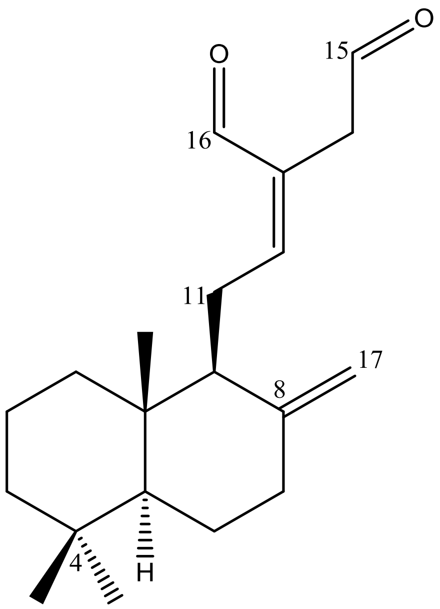

Compound 1

^{1}H NMR spectra of compound 1:

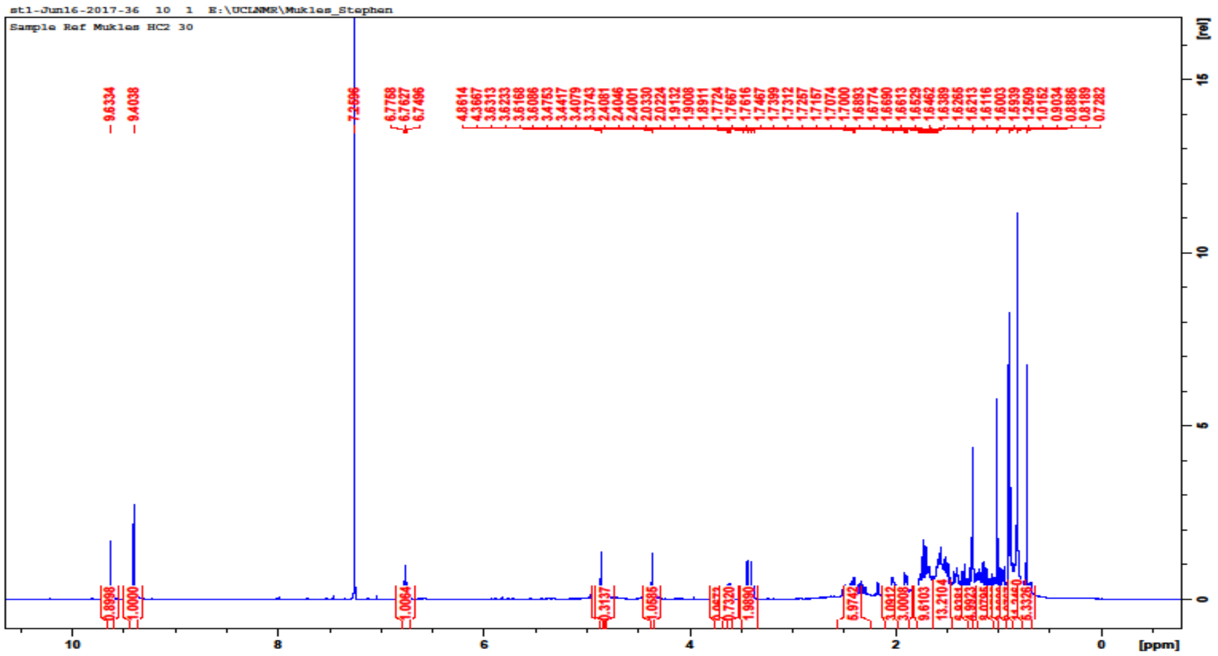

^{1}H NMR spectra (expanded) of compound 1:

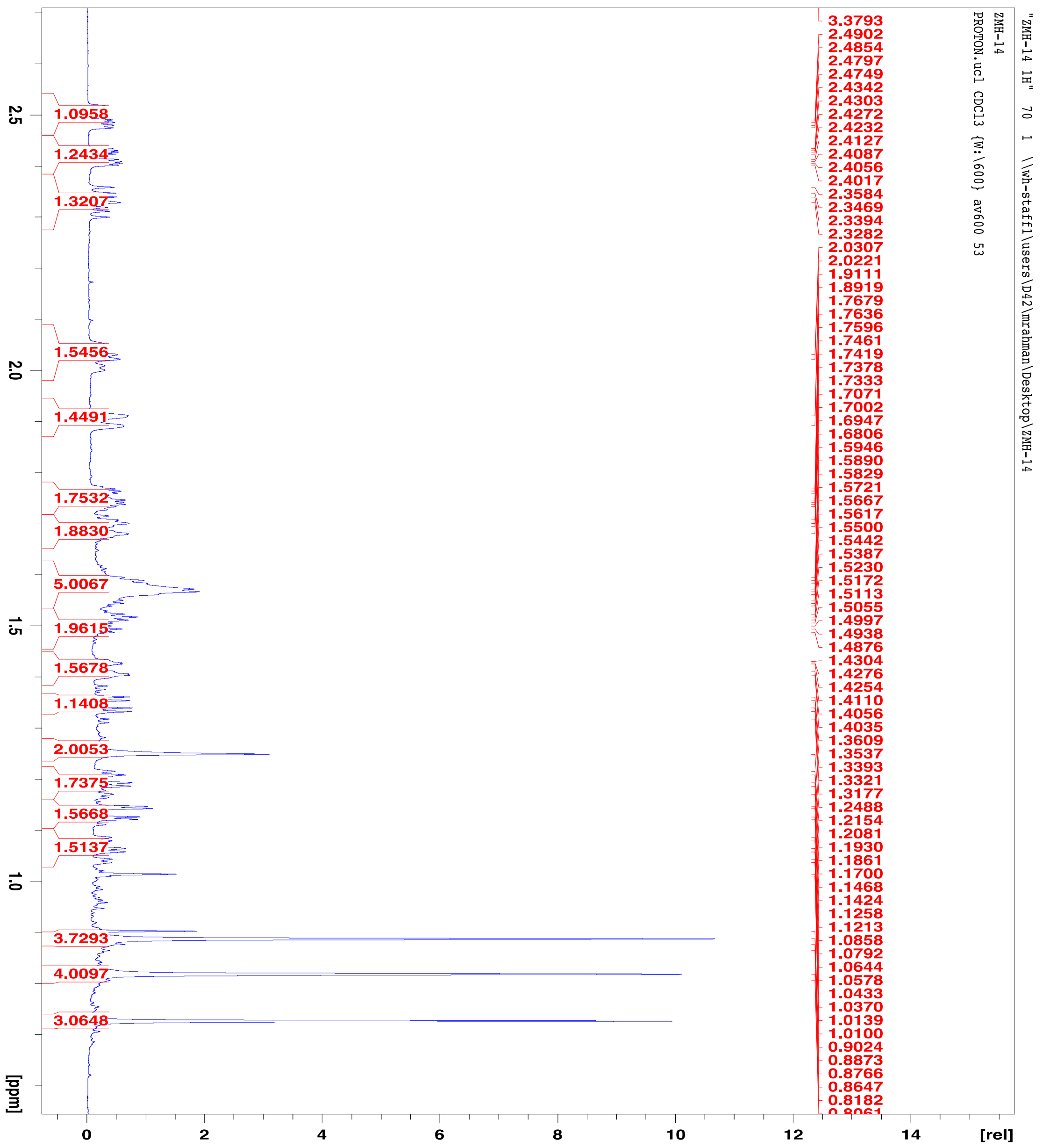

^{1}H NMR spectra (expanded) of compound 1:

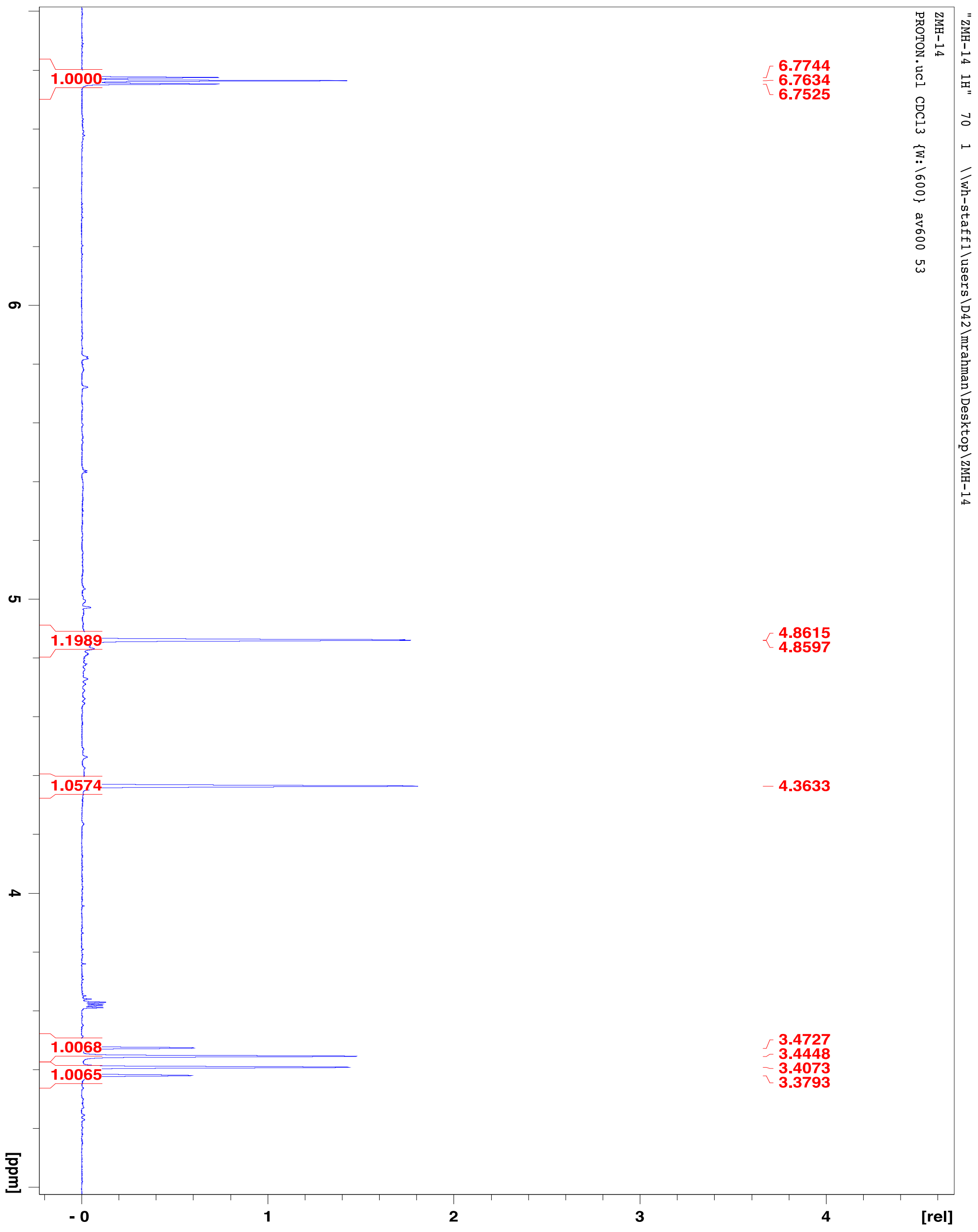

^{13}C NMR spectra of compound 1:

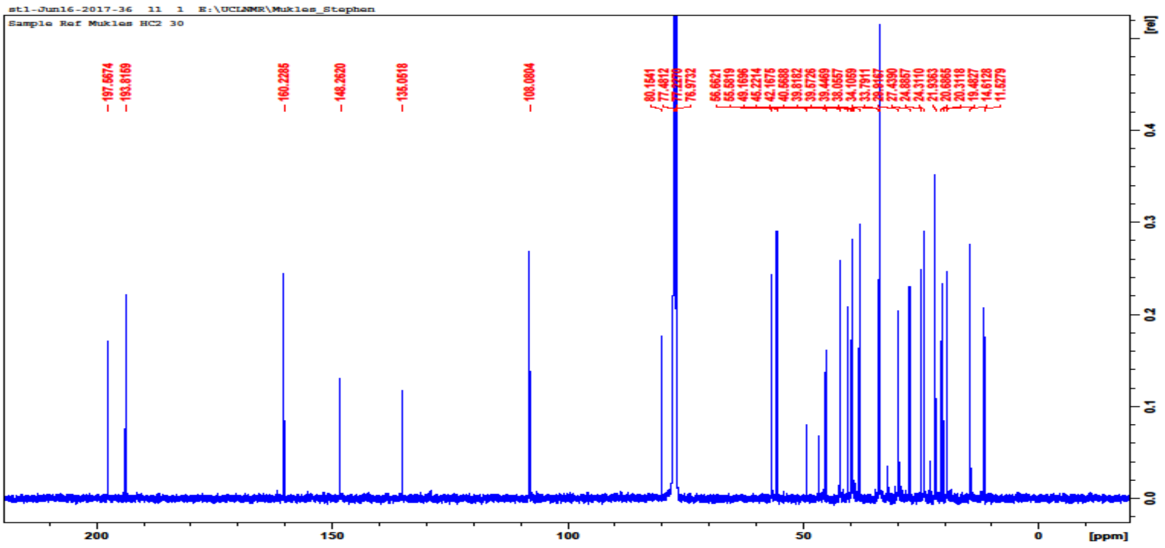

HSQC NMR spectra of compound 1:

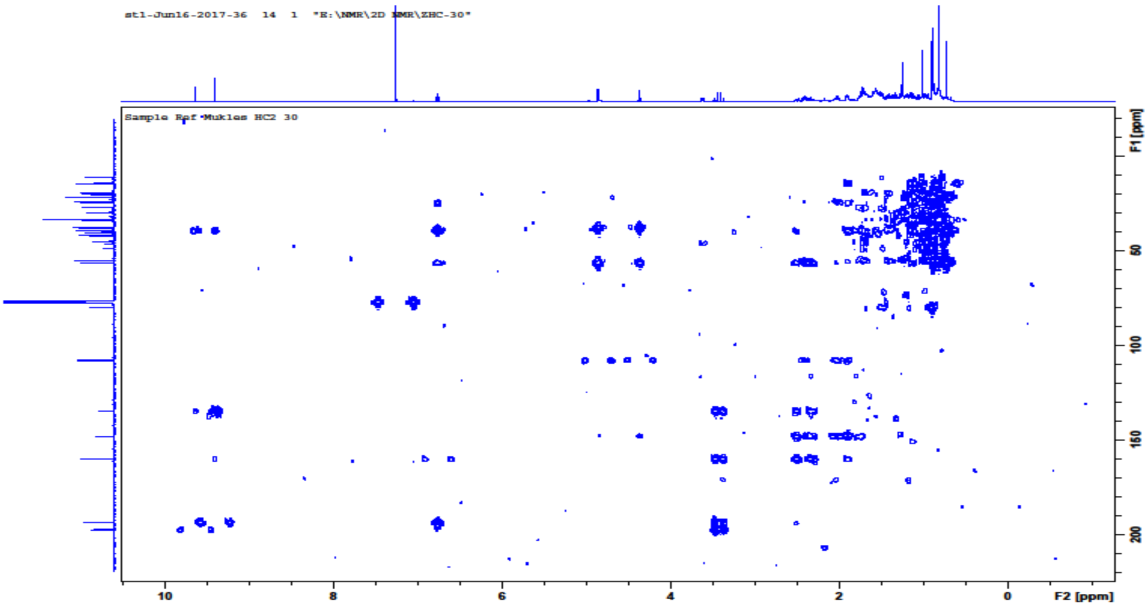

HMBC NMR spectra of compound 1:

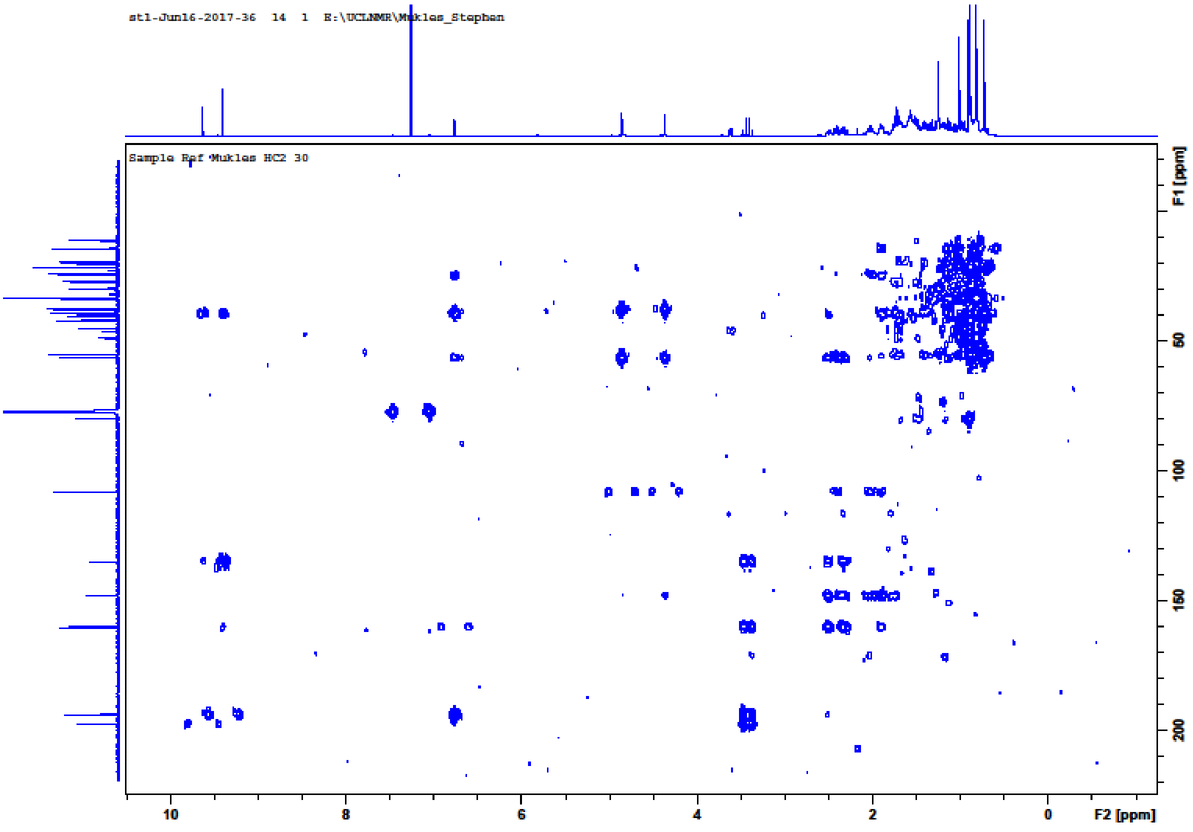

HRMS (High Resolution Mass Spectroscopy) of compound 1:

The high resolution of mass spectroscopy showed the $[M+H]^+$ at m/z 303.23122 (calculated for $C_{20}H_{31}O_2$, at 303.224580) corresponding to the molecular formula of 1 as $C_{20}H_{30}O_2$.

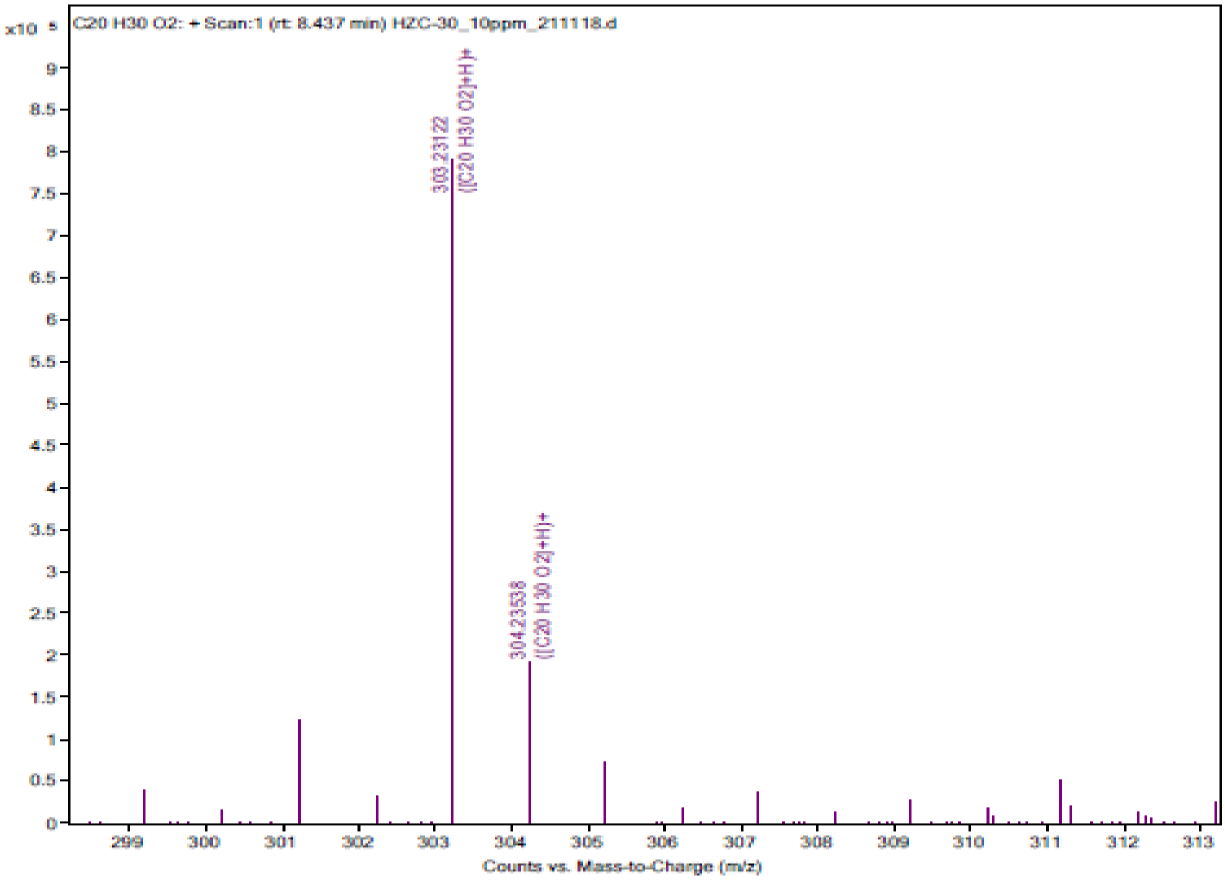

^{1}H and ^{13}C NMR spectra of compound 2:

4.63 (d, *J*= 7.5 Hz, 1H, H-1), 5.20 (d, *J*= 7.5Hz, 1H, H-3), 2.20 (m, 1H, H-4a), 2.24 (m, 1H, H-4b); 2.35 (m, 2H, H-5), 4.82, dd, *J*= 10.2, 4.4 Hz, 1H, H-7), 1.87 (m, 1H, H-8a), 2.32 (m, 1H, H-8b); 5.23 (d, *J*= 16.2 Hz, 1H, H-10), 5.56 (dd, *J*= 16.2, 7.5 Hz, 1H, H-11), 1.65 (s, 3H, H-12), 1.43 (s, 3H, H-13), 1.04 (s, 3H, H-14) and 1.06 (s, 3H, H-15).

78.8 (C-1), 142.2 (C-2), 124.8(C-3), 24.4 (C-4), 39.5 (C-5), 133.2 (C-6), 125.0 (C-7), 42.4 (C-8), 37.3 (C-9), 139.5 (C-10), 131.2 (C-11), 12.8 (C-12), 15.3 (C-13), 24.9 (C-14) and 29.9 (C-15).

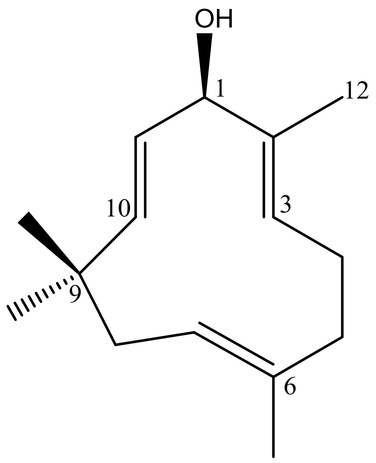

Compound 2

^{1}H NMR spectra of **compound 2**

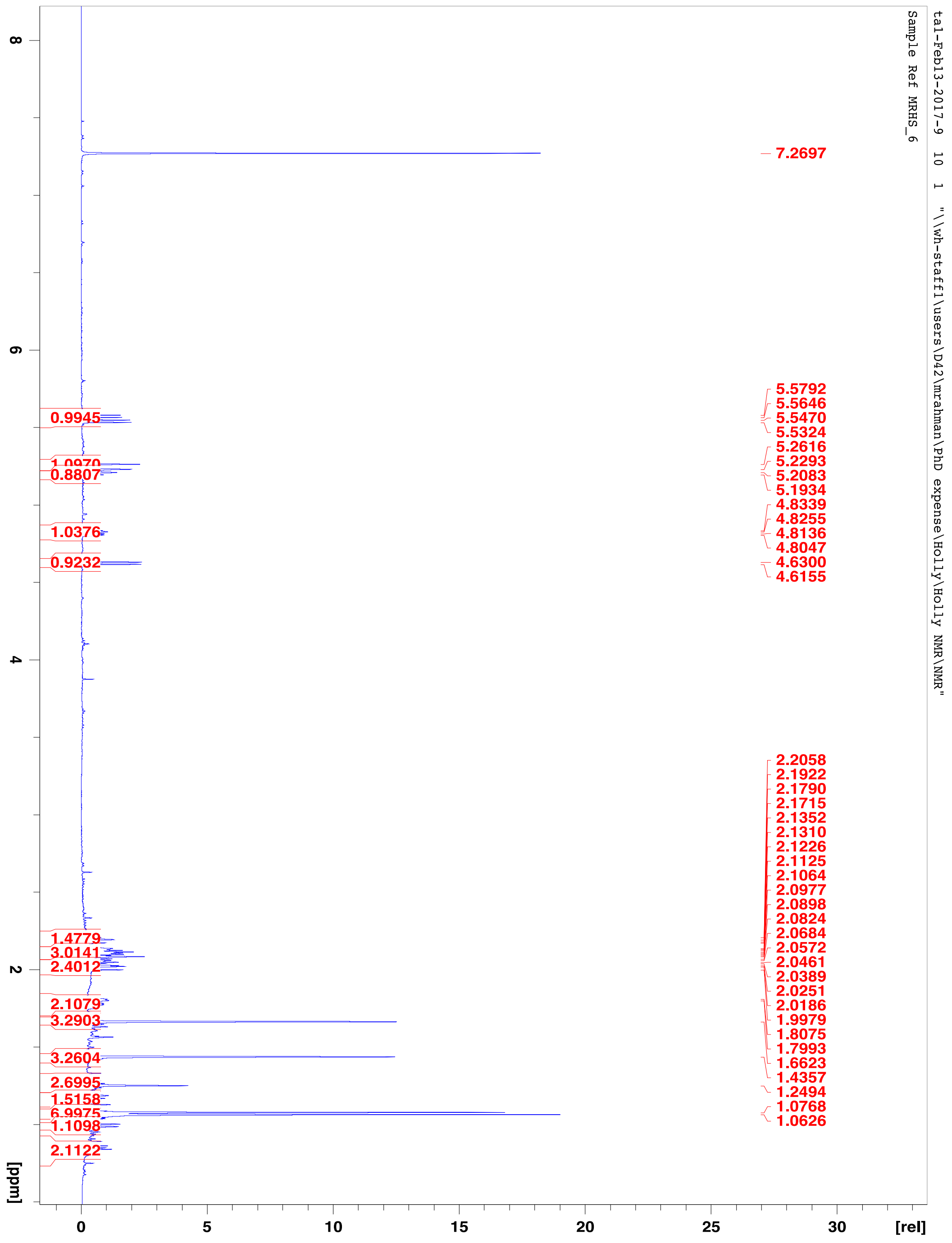

^{1}H NMR spectra (expanded) of **compound 2**

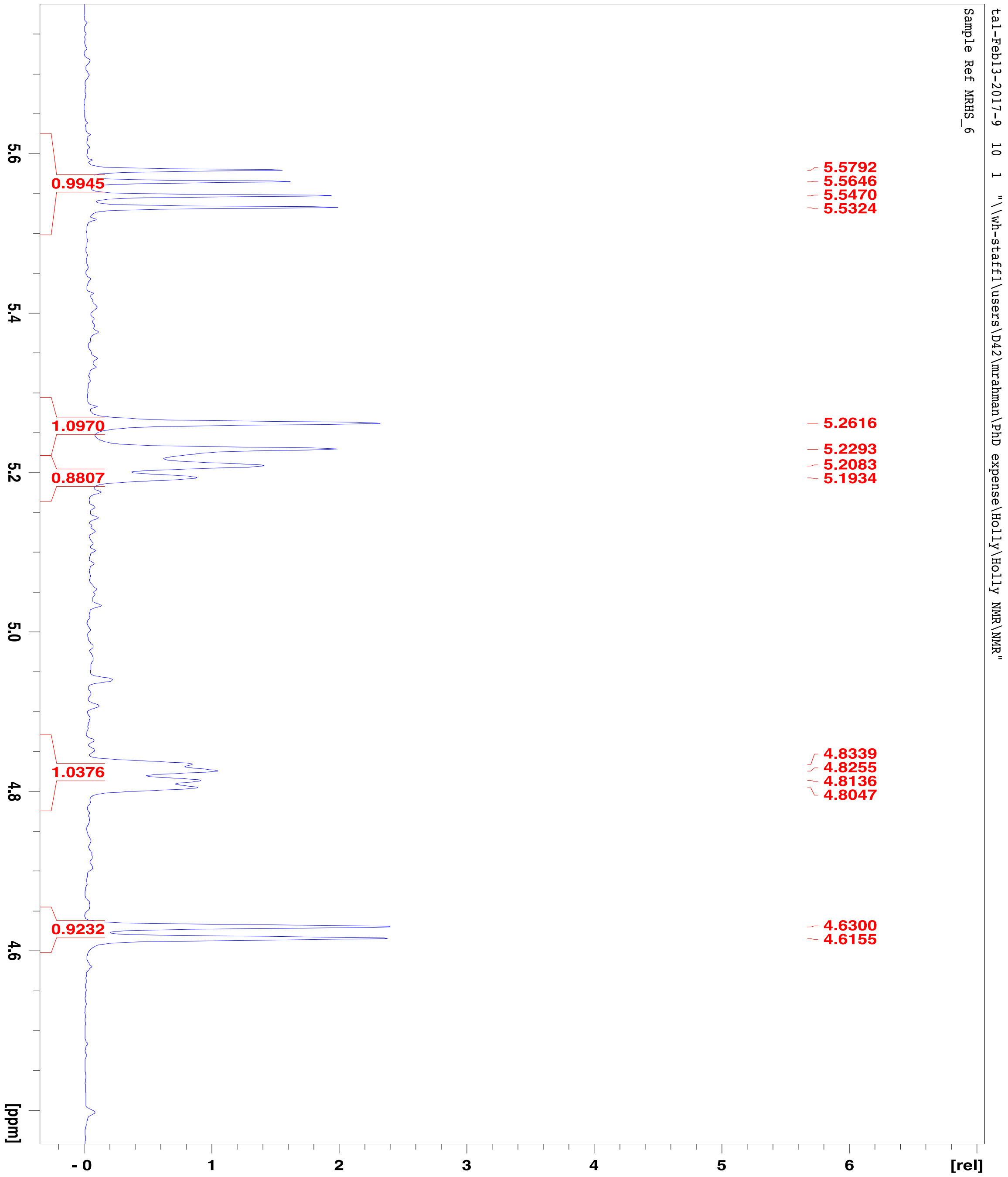

^{13}C NMR spectra of compound 2

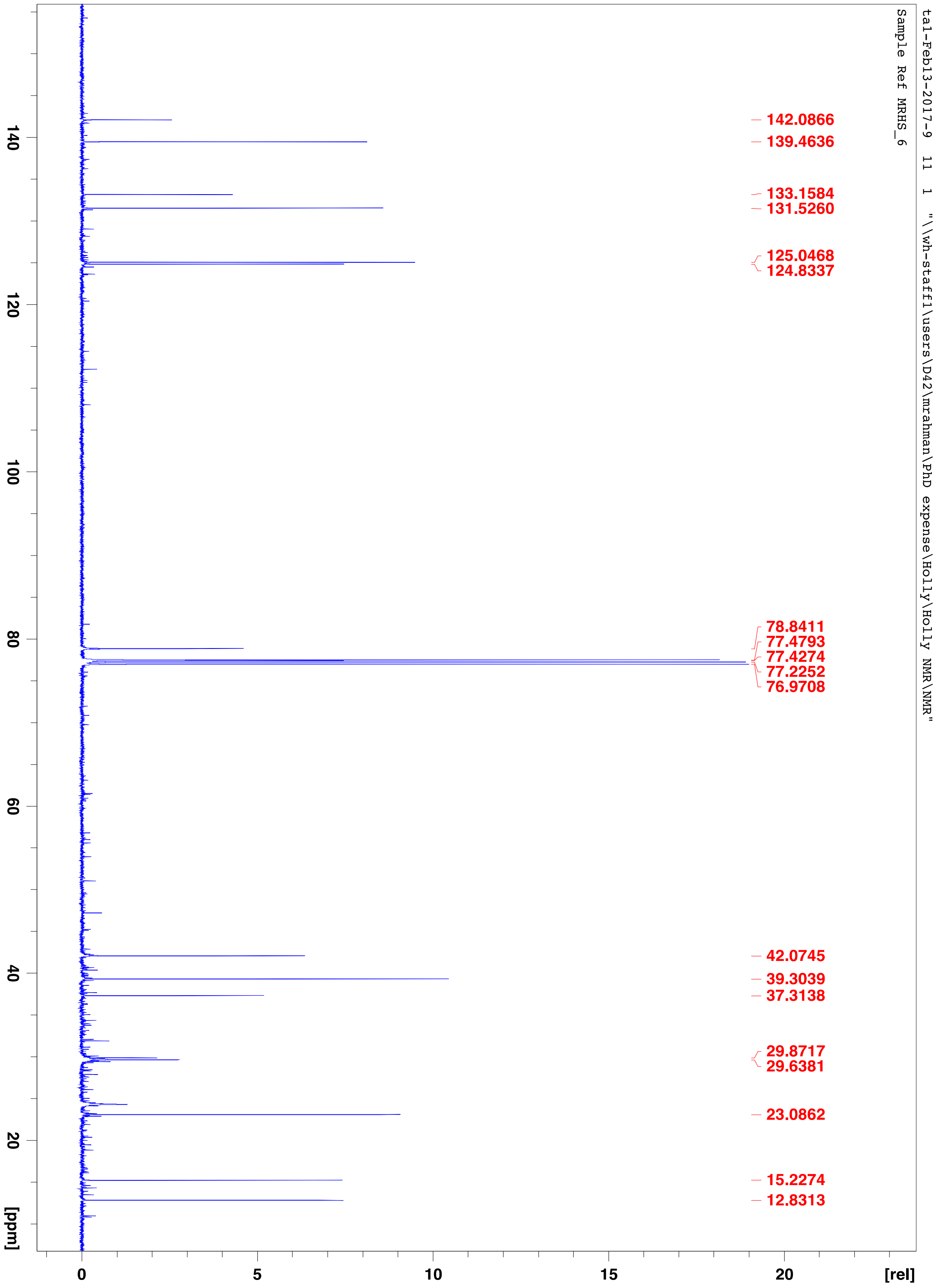

COSY of compound 2:

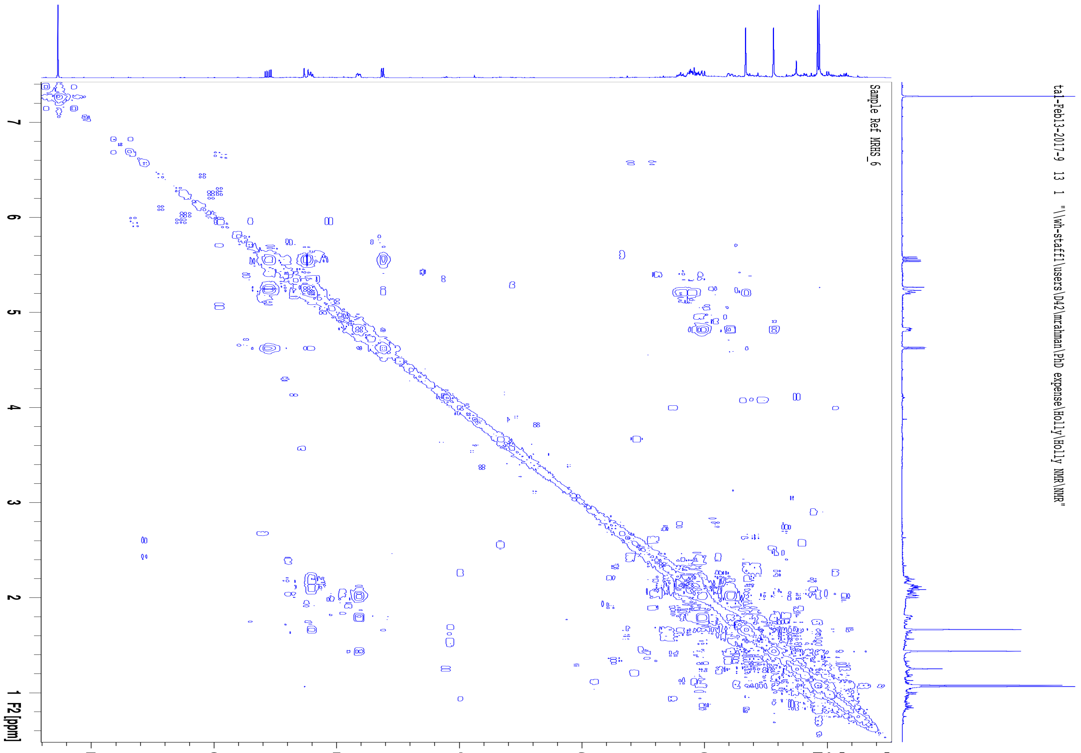

HSQC of compound 2:

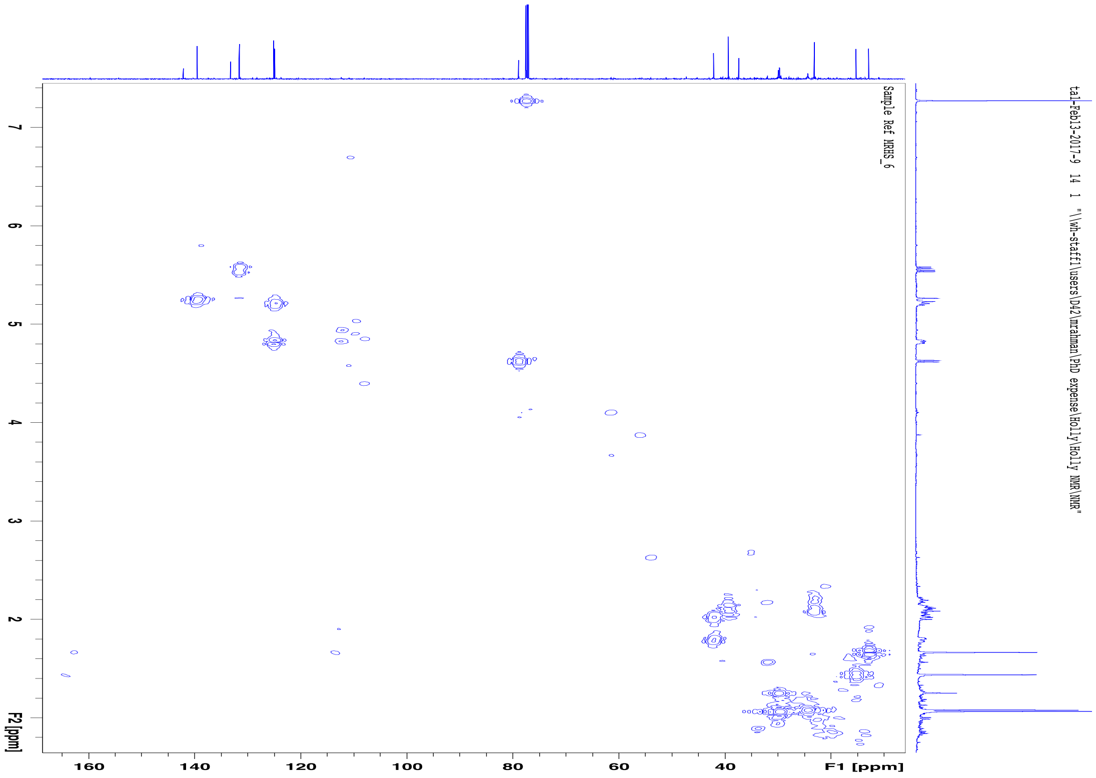

HMBC of compound 2:

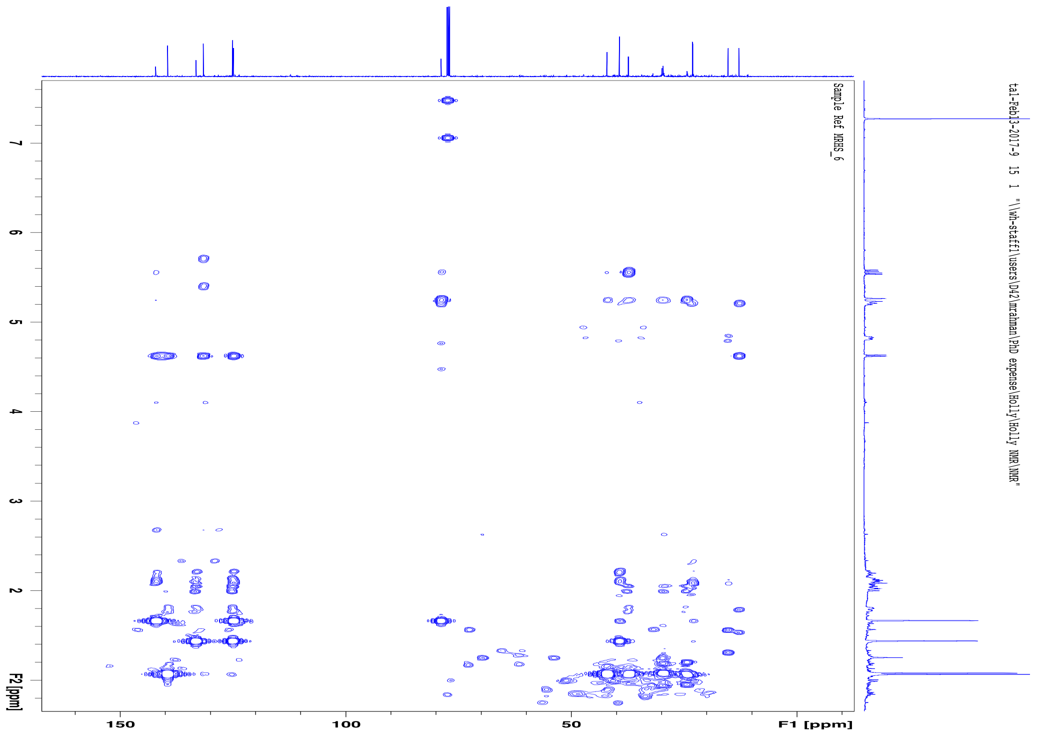

HRMS (High Resolution Mass Spectrometry) of compound 2:

The high resolution of mass spectroscopy showed the $[M+H]^+$ at m/z 221.18956 (calculated for $C_{15}H_{25}O$, at 221.19054) corresponding to the molecular formula of 2 as $C_{15}H_{24}O$.

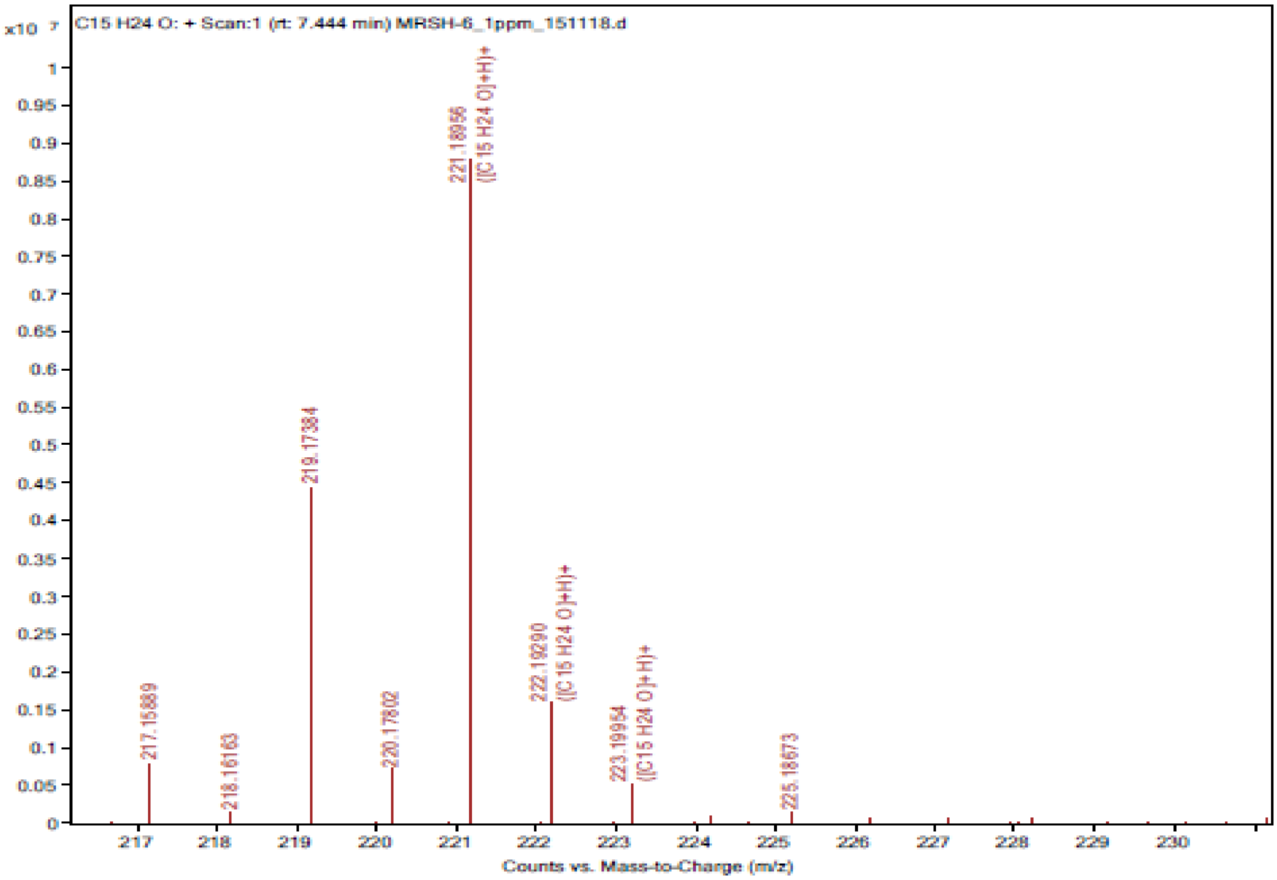

Figure S1: Antibacterial screening of compounds 1-3 and norfloxacin against MRSA strains:

(1= Compound 1; 2= Compound 2; 3=compound 3 and Nor= Norfloxacin)

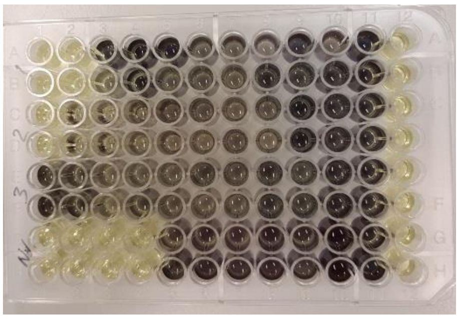

MICs against ATCC 25923

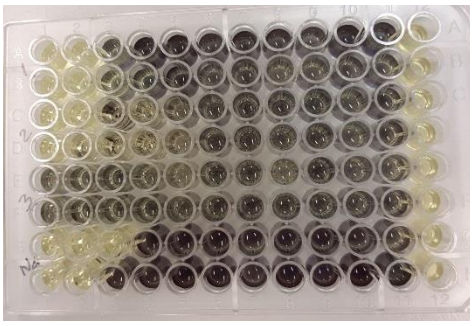

MICs against SA1199B

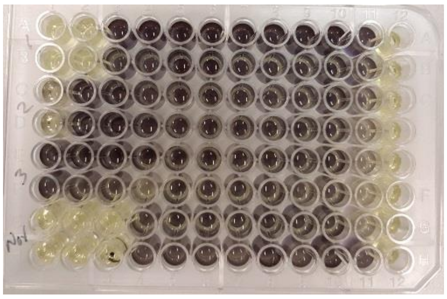

MICs against EMRSA 15

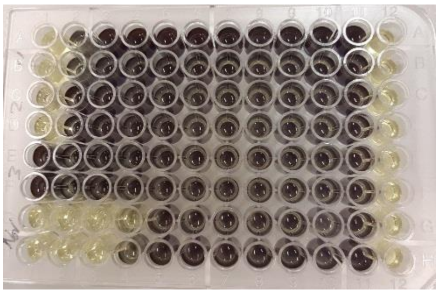

MICs against MRSA 274829

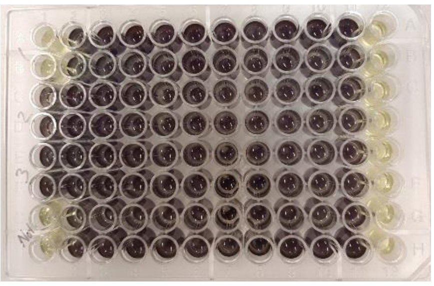

MICs against MRSA 340702

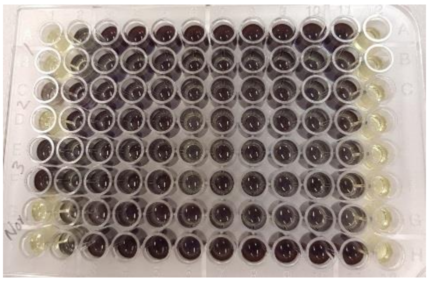

MICs against XU212
